# ﻿*Paracorymbiglomus* gen. nov., *Diversisporaconica* sp. nov., and new combinations in Diversisporaceae (Glomeromycota)

**DOI:** 10.3897/mycokeys.117.148052

**Published:** 2025-05-05

**Authors:** Janusz Błaszkowski, Bruno Tomio Goto, Szymon Zubek, Paweł Milczarski, Ryszard Malinowski, Piotr Niezgoda, Tomasz Błaszkowski

**Affiliations:** 1 Department of Environmental Management, West Pomeranian University of Technology in Szczecin, Słowackiego 17, PL–71434 Szczecin, Poland; 2 Departamento de Botânica e Zoologia, Universidade Federal do Rio Grande do Norte, Campus Universitário, 59078–900, Natal, RN, Brazil; 3 Institute of Botany, Faculty of Biology, Jagiellonian University, 30–387, Kraków, Poland; 4 Department of Genetic, Plant Breeding & Biotechnology, West Pomeranian University of Technology in Szczecin, Słowackiego 17, PL–71434 Szczecin, Poland; 5 Department of General and Oncological Surgery, Pomeranian Medical University in Szczecin, Szczecin, Poland

**Keywords:** Arbuscular mycorrhizal fungi, morphology, nuc rDNA, phylogenetic taxonomy, *rpb1*

## Abstract

The paper presents the results of morphological studies, as well as comparisons and phylogenetic analyses of sequences of the 45S (= 18S-ITS-28S) nuc rDNA region and the *rpb1* gene (when available) of four arbuscular mycorrhizal fungi (AMF) of the phylum Glomeromycota. These fungi were (i) an informally named isolate 449, suspected of being an undescribed species of the genus *Diversispora*, and (ii) three *Corymbiglomus* species in the family Diversisporaceae. The studies confirmed the novelty of isolate 449 in *Diversispora* and showed that *Corymbiglomus* contains only *C.corymbiforme*, while *C.globiferum* and *C.pacificum* should be transferred to a separate genus sister to *Corymbiglomus*. Consequently, isolate 449 was described as *Diversisporaconica***sp. nov.**, *C.globiferum*, and *C.pacificum* were placed in *Paracorymbiglomus***gen. nov.** and renamed *P.globiferum***comb. nov.** and *P.pacificum***comb. nov.**

## ﻿Introduction

Diversisporales is one of the six orders of the phylum Glomeromycota, which includes arbuscular mycorrhizal fungi (AMF; Redecker et al. 2013; Błaszkowski et al. 2022b; Hyde et al. 2024; Wijayawardene et al. 2024). Diversisporales consists of four families, including Diversisporaceae (Redecker et al. 2013). This family contains five genera, among which are *Corymbiglomus*, *Diversispora*, and *Redeckera*, which are the main subjects of this article.

With the exception of *D.otospora* and *D.tricispora*, which produce otosporoid and entrophosporoid spores, respectively, sensu Oehl et al. (2011a, b), the common feature shared by the remaining members of these three genera is the formation of spores blastically at the tips of subtending hyphae. This mode of spore formation occurs in *Glomusmacrocarpum*, the type species of Glomeromycota (Schüßler and Walker 2010). Therefore, Morton and Redecker (2001) called the spores thus formed glomoid. However, Oehl et al. (2011a, b) found that glomoid spores sensu Morton and Redecker (2001) may differ significantly in the subcellular spore structure and properties of the spore subtending hypha. In spores of *Corymbiglomus* species, the differences have not been clearly defined (Błaszkowski 2012; Medina et al. 2014; Błaszkowski et al. 2019) and, consequently, contributed to the uncertain classification of members of this genus and prevented clear definition of morphological boundaries between species of *Corymbiglomus*, *Diversispora*, and *Redeckera*.

Currently, *Corymbiglomus* comprises three species: *C.corymbiforme* and *C.globiferum*, originally described in the genus *Glomus* (Błaszkowski 1995; Koske and Walker 1986), and *C.pacificum* (Medina et al. 2014; Błaszkowski et al. 2019). Medina et al. (2014) considered the subcellular structure of spores in these species to consist of two spore walls. In contrast, Błaszkowski (2012) and Błaszkowski et al. (2019) described *C.corymbiforme* spores as having a single spore wall. Furthermore, Błaszkowski et al. (2019) considered *C.globiferum* and *C.pacificum* to also produce single-walled spores, with three and four layers, respectively. However, this conclusion was based on analyses of a small number of specimens permanently mounted on microscope slides. Furthermore, the location of spore wall layer 3 of *C.globiferum* was difficult to determine unequivocally because of the dense hyphal mantle covering the spore surface and rendering spore wall layer 3 almost invisible at the spore base.

As for *Diversispora* and *Redeckera*, except for *D.nevadensis* and *D.tricispora*, Oehl et al. (2011a, b) unequivocally showed the uniqueness of features of the subcellular spore structure and spore subtending hyphae of members of these genera and named these single-walled spores diversisporoid.

Literature data suggest that the phylogenies of *Corymbiglomus* and *Redeckera* species are uncertain. Błaszkowski et al. (2019) concluded that the natural relationship of *C.corymbiforme* to *C.globiferum* and *C.pacificum* is not only distant but also weak and that *C.corymbiforme* could occupy an autonomous generic clade. Phylogenetic analyses by Tedersoo et al. (2024) nested *C.corymbiforme* within the genus *Redeckera*, whose sister clade was inhabited by *C.globiferum*.

We have grown in culture an AM fungus informally named isolate 449 and obtained its genetic sequences. Preliminary comparisons and phylogenetic analyses of these sequences with sequences available in public databases suggested that isolate 449 is an undescribed *Diversispora* species.

The aims of the subsequent analyses described in this paper were to (i) verify the morphological similarities of *C.corymbiforme*, *C.globiferum*, and *C.pacificum*; (ii) resolve ambiguities in the phylogenies of *Corymbiglomus* species and *C.corymbiforme* relative to *Redeckera* species; and (iv) characterize the morphology and phylogeny of isolate 449.

## ﻿Materials and methods

### ﻿Origin of study material

Isolate 449 was originally found in a trap pot culture inoculated with a mixture of rhizosphere soil and root fragments of *Ammophilaarenaria* (L.) Link. This plant colonized the Mediterranean dunes of the beach Voidokoilia (36°57'N, 21°39'E) located on the Peloponnese Peninsula, Greece. The soil sample was collected by J. Błaszkowski on September 8, 2015. Data about the climate and soil chemical properties of the sampled site are in Błaszkowski et al. (2019). Methods used to establish trap and single-species cultures, growing conditions, as well as methods of spore extraction and staining of mycorrhizal structures, were as those described by Błaszkowski et al. (2012). Single-species pot cultures were inoculated with five to ca. 15 spores of uniform morphology.

The origin of *Corymbiglomus* species and *R.megalocarpum* was given in Błaszkowski (2012) and Błaszkowski et al. (2019). *Corymbiglomusglobiferum* was also analyzed using descriptions and illustrations available on the INVAM website (https://invam.ku.edu/). In addition, specimens of *R.fragile* (Trappe 3316, 5429), *R.fulvum* (Garcia 2149, Trappe 3312, 9927), and *R.pulvinatum* (Trappe 9875, 13439, 16696) obtained from Dr. James M. Trappe were analyzed.

### ﻿Microscopy and nomenclature

Morphological features of spores as well as phenotypic and histochemical features of the spore wall and subtending hyphal wall layers of Isolate 449 were characterized from approximately 100 spores mounted in water, lactic acid, polyvinyl alcohol/lactic acid/glycerol (PVLG, Omar et al. 1979), and a mixture of PVLG and Melzer’s reagent (1:1, v/v). The spores were from a trap culture and three single-species cultures. Verifications of the morphologies of *Corymbiglomus* species and comparisons of these morphologies with those of *Redeckera* species were carried out using the study material and data, whose origin was given above. Spores for study and photography were prepared as described in Błaszkowski (2012) and Błaszkowski et al. (2012). The types of spore wall layers were defined by Błaszkowski (2012) and Walker (1983). Color names were from Kornerup and Wanscher (1983). Nomenclature of fungi and the authors of fungal names are from the Index Fungorum website https://indexfungorum.org. The term “glomerospores” was used for spores produced by AMF as proposed by Goto and Maia (2006).

The holotype of the new species was deposited at ZT Myc (ETH Zurich, Switzerland), and its isotypes in the Laboratory of Plant Protection, Department of Environmental Management (LPPDEM), West Pomeranian University of Technology in Szczecin, Poland. In all specimens, spores were permanently mounted in PVLG and a mixture of PVLG and Melzer’s reagent (1:1, v/v) on slides.

### ﻿DNA extraction, PCR, cloning, and DNA sequencing

Genomic DNA of Isolate 449 was extracted from eight samples, each with ca. 5–20 spores coming from single-species cultures. The method of processing the spores prior to PCR, conditions, and primers used for PCR to obtain 45S sequences of the isolate were as those described by Krüger et al. (2009) and Błaszkowski et al. (2021). Numerous attempts to obtain *rpb1* sequences of Isolate 449 performed with the primers by Stockinger et al. (2014) and those presented in Błaszkowski et al. (2022a) failed. Cloning and sequencing of PCR products to obtain 45S sequences were performed following protocols described by Błaszkowski et al. (2021). The sequences were deposited in GenBank (PV345770–PV345773).

### ﻿Phylogenetic analyses

Preliminary comparisons of 45S sequences of Isolate 449 with sequences of this region or its part, available in GenBank, showed that Isolate 449 belongs to *Diversispora*. To find the position and taxonomic status of this fungus and clarify the doubts regarding *Corymbiglomus* species, described in “Introduction,” three alignments, 45S, *rpb1*, and 45S+*rpb1*, were prepared using MAFFT 7 with the E-INS-i option (Katoh et al. 2019).

**Figure 1.**
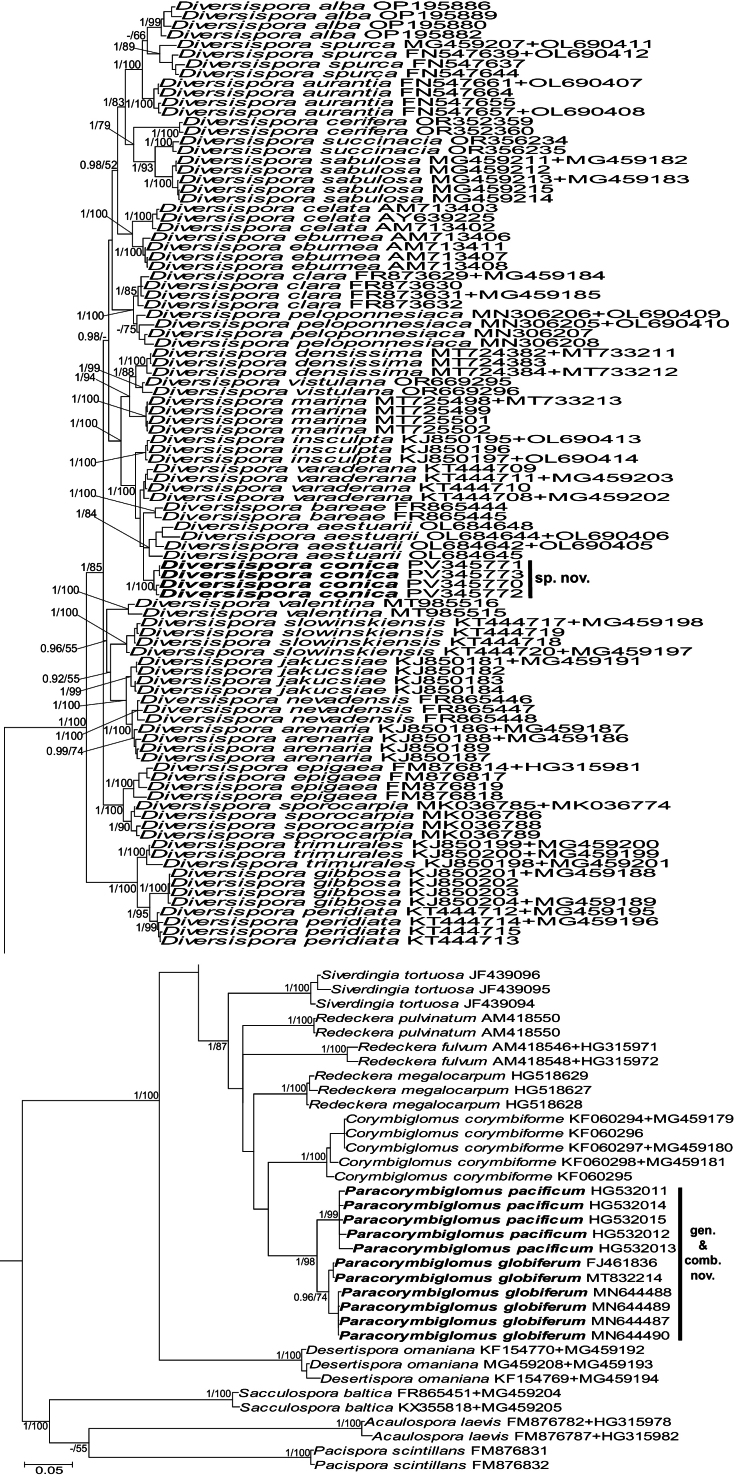
50% majority-rule consensus tree from the Bayesian analysis of the 45S+*rpb1* alignment with sequences of *Diversisporaconica* (= isolate 449), *Corymbiglomuscorymbiforme*, *Paracorymbiglomusglobiferum*, *P.pacificum*, 32 other species of Diversisporaceae, as well as *Acaulosporalaevis*, *Pacisporascintillans*, and *Sacculosporabaltica* serving as outgroup. The new species and genus are in bold font. The Bayesian posterior probabilities ≥ 0.90 and ML bootstrap values ≥ 50% are shown near the branches, respectively. Bar indicates a 0.05 expected change per site per branch.

The ingroup of the 45S alignment were sequences of the 45S rDNA nuc region or part thereof that characterized all sequenced species of *Corymbiglomus*, *Diversispora*, *Redeckera*, and the remaining genera of Diversisporaceae (Suppl. material 1). The outgroup were sequences of species of *Acaulospora*, *Pacispora*, and *Sacculospora*. In exploratory phylogenetic analyses with sequences from representatives of all genera of Diversisporales, these three genera were shown to be most closely related to the ingroup taxa (data not shown). The ingroup and outgroup of the *rpb1* alignment were sequences of all species of the 45S alignment that were provided with available sequences of the *rpb1* gene (Suppl. material 2). The 45S+*rpb1* alignment contained all sequences of the 45S alignment concatenated with sequences of the *rpb1* alignment (Fig. 1). The 45S and *rpb1* alignments were divided into five (18S, ITS1, 5.8S, ITS2, 28S) and three (exons 4 and 5, intron 4) partitions, respectively, and the 45S+*rpb1* alignment into eight partitions.

To reconstruct the phylogenetic positions of Isolate 449 and the remaining major taxa studied here, these three alignments were subjected to Bayesian inference (BI) and maximum likelihood (ML) phylogenetic analyses, performed via CIPRES Science Gateway 3.1 (Miller et al. 2010). In both BI and ML analyses, GTR+I+G was used as a nucleotide substitution model for each nucleotide partition, as suggested by Abadi et al. (2019).

The BI reconstructions were made based on four Markov chains run over one million generations in MrBayes 3.2 (Ronquist et al. 2012), sampling every 1,000 generations, with a burn-in at 30% sampled trees. The ML phylogenetic tree inferences were performed with RAxML-NG 1.0.1 (Kozlov et al. 2019), using a maximum likelihood/1000 bootstrapping run and an ML estimated proportion of invariable sites and base frequencies. The alignments and tree files were deposited as supplementary materials. Clade and node supports were considered strong, moderate, and marginal when BI and ML support values were 0.98–0.99 and 81–99%, 0.96–0.97 and 71–80%, and 0.95 and 70%, respectively. The phylogenetic trees were visualized and edited in FigTree ver. 1.4.4 (http://tree.bio.ed.ac.uk/software/figtree/) and MEGA6 (Tamura et al. 2013).

The percentage sequence divergences of the fungi analyzed here were calculated in BioEdit (Hall 1999). All comparisons were performed on sequences of the same length.

To detect possible other findings of Isolate 449, its 45S sequences were used as queries in BLASTn to find environmental sequences of potentially identical species from GenBank. The sequences were selected according to the percentage of identity > 96%. Their likely identity was then verified in BI and ML analyses of the alignments with 45S sequences.

## ﻿Results

### ﻿General data and phylogeny

The alignments analyzed contained four newly obtained sequences of the 45S region. The numbers of analyzed sequences and species/isolate, as well as the numbers of base pairs, variables, and parsimony informative sites of each of the alignments, are presented in Table 1.

**Table 1.** Characteristics of the sequence alignments analyzed.

Name of alignment	No. of sequences	No. of fungal species	No. of base pairs	No. of variable sites	No. of parsimony informative sites
45S	133	39	1839	909	792
*rpb1*	48	24	809	290	267
45S+*rpb1*	133	39	2648	1199	1059

The topologies of the 45S+*rpb1* and 45S trees were similar (Fig. 1, Suppl. material 1). Small and insignificant differences in the topology of the *rpb1* tree compared to the topologies of the 45S+*rpb1* and 45S trees resulted from the lack of *rpb1* sequences of 15 of the 39 analyzed species (Suppl. material 2).

In the 45S+*rpb1* tree, the ingroup sequences were distributed in three major clades inhabited by (i) *Diversispora* species, (ii) *Corymbiglomus*, *Redeckera*, and *Sieverdingia* species, and (iii) *Desertisporaomaniana* (Fig. 1). Each of these clades received full or strong BI and ML support, except for the second clade, whose ML support was moderate. The ingroup of the 45S tree consisted of two major clades with sequences of (i) *Diversispora* species and (ii) species of the other genera listed above (Suppl. material 1). BI and ML supports for the first clade were full, while none of the BI and ML analyses supported the second clade. In both analyses, the entire ingroup was fully supported.

In the 45S+*rpb1* and 45S trees, *Corymbiglomus* species sequences grouped in a clade sister to that with *R.megalocarpum* sequences (Fig. 1, Suppl. material 1). None of the BI and ML analyses supported the node connecting these two clades.

In both trees, *C.globiferum* and *C.pacificum* sequences were placed in sister subclades in a position sister to the clade with *C.corymbiforme* sequences (Fig. 1, Suppl. material 1). Both the *C.corymbiforme* clade and the *C.globiferum* plus *C.pacificum* clade were fully supported by BI analyses and fully or strongly supported by ML analyses. Neither analysis supported the node connecting the *C.corymbiforme* clade with the *C.globiferum* plus *C.pacificum* clade.

Both BI and ML analyses showed that *R.fulvum* and *R.pulvinatum* were unrelated to each other and to *R.megalocarpum* (Fig. 1, Suppl. material 1).

In both trees, Isolate 449 was found to be a member of a three-species clade. In the 45S+*rpb1* tree, the sister species of Isolate 449 was *D.aestuarii* (Fig. 1), while in the 45S tree the sister species was *D.bareae* (Suppl. material 1). Isolate 449 received full BI and ML support in these trees. However, neither analysis supported this three-species clade.

When sequences of the 45S region or part thereof were compared, the genetic distances between (i) Isolate 449 versus *D.aestuarii*, (ii) Isolate 449 vs. *D.bareae*, (iii) *C.corymbiforme* vs. *C.pacificum*, (iv) *C.corymbiforme* vs. *C.globiferum*, (v) *C.globiferum* vs. *C.pacificum*, (vi) *C.corymbiforme* vs. *R.megalocarpum*, (vii) *C.corymbiforme* vs. *R.fulvum*, and (viii) *C.corymbiforme* vs. *R.pulvinatum* were (i) 5.6–6.2%, (ii) 3.8–4.6%, (iii) 12.4–14.0%, (iv) 11.6–15.1%, (v) 4.4–5.5%, (vi) 10.4–12.8%, (vii) 42.4–43.0%, and (viii) 41.9–43.4%, respectively. Comparisons of *rpb1* sequences of sister or closely related species shown in the *rpb1* tree (Suppl. material 2) indicated that the genetic divergences between (i) *C.corymbiforme* vs. *R.fulvum*, (ii) *De.omaniana* vs. *R.fulvum*, (iii) *De.omaniana* vs. *D.sabulosa*, and (iv) *Diversispora* species were (i) 7.4–7.9%, (ii) 17.8–18.5%, (iii) 17.0–17.3%, and (iv) 0.6–3.5%, respectively.

### ﻿Taxonomy

The phylogenetic analyses and sequence comparisons of *Corymbiglomus* and *Redeckera* species and Isolate 449 showed that (i) the placement of *C.corymbiforme* in the genus *Corymbiglomus* is correct, (ii) *C.globiferum* and *C.pacificum* should be transferred to a new genus, sister to *Corymbiglomus*, and (iii) Isolate 449 is a new *Diversispora* species. Consequently, (i) *C.globiferum* and *C.pacificum* were transferred to *Paracorymbiglomus* gen. nov. with *P.globiferum* comb. nov. and *P.pacificum* comb. nov. and (ii) Isolate 449 was described as *D.conica* sp. nov.

### ﻿Descriptions of a new genus, new combinations, emended *Corymbiglomus*, and a new species

#### ﻿ Paracorymbiglomus

Taxon classificationFungiDiversisporalesDiversisporaceae

Błaszk., Niezgoda & B.T.Goto gen. nov.

04B4A80D-C0AD-5CE8-BCAF-755A1CB2AF52

858391

**Figure 2.**
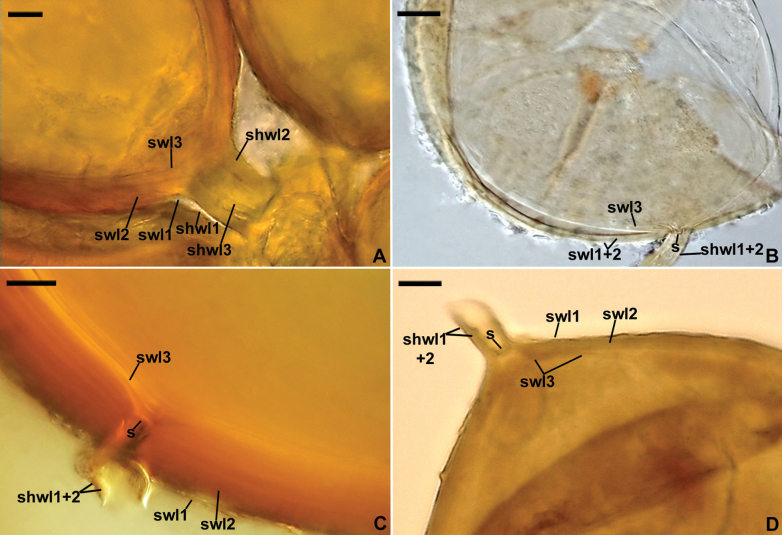
*Corymbiglomus* versus *Paracorymbiglomus***A** subtending hyphal wall layers (shwl) 1–3 continuous with spore wall layers (swl) 1–3 of *C.corymbiforme***B, C, D** shwl 1 and 2 continuous with only swl1 and 2 of *P.globiferum* (**B, C**) and *P.pacificum* (**C**); in **B** the invagination of swl3 forms a septum (s) occluding the spore pore; in **C** and **D** the spore pore is occluded by the invagination of swl3 and a septum (s) connecting the inner surfaces of swl2 forming the subtending hyphal lumen.

Figs 1 , 2 , Suppl. material 1

##### Etymology.

Latin, *Paracorymbiglomus*, referring to the great morphological similarity and close phylogenetic relationship of this genus to *Corymbiglomus*.

##### Type genus.

*Paracorymbiglomusglobiferum* (Koske & C. Walker) Błaszk., Niezgoda & B.T.Goto comb. nov. MycoBank No: 858393

##### Basionym.

*Glomusglobiferum* Koske & C. Walker.

##### Synonym.

*Corymbiglomusglobiferum* (Koske & C. Walker) Błaszk. & Chwat.

##### Other species.

*Paracorymbiglomuspacificum* (Oehl, J. Medina, P. Cornejo, Sánchez-Castro, G.A. Silva & Palenz.) Błaszk., Niezgoda & B.T.Goto comb. nov. MycoBank No: 858394

***Basionym***: *Corymbiglomuspacificum* Oehl, J. Medina, P. Cornejo, Sánchez-Castro, G.A. Silva & Palenz.

##### Diagnosis.

Differs from *Corymbiglomus*, the sister monospecific genus with *C.corymbiforme* (Fig. 1, Suppl. material 1), in (i) the site of initiation of formation of the innermost spore wall layer (Fig. 2), (ii) the composition of the spore subtending hyphal wall (Fig. 2), (iii) the mode of formation and morphology of the hyphal mantle covering the spore surface, and (iv) the nucleotide composition of sequences of the 45S nuc rDNA region (see “Discussion” for details).

##### Genus description.

Spores hypogeous, formed singly, occasionally in clusters. Spores light- to dark-colored, globose to subglobose, 85–320 µm diam, rarely ellipsoid to irregular, with one, rarely two to three subtending hyphae. Subcellular structure of spores composed of one spore wall containing three or four layers. Outermost spore wall layer, forming the spore surface, covered or not covered by a hyphal mantle. Innermost spore wall layer laminate, semi-flexible, hyaline, 2.0–4.8 µm thick, usually loosely associated with and readily separating from the inner surface of the penultimate spore wall layer in crushed spores, except at the spore base, where it usually forms an inseparable association due to having a small process recessed into the spore subtending hyphal lumen to at most the spore base. Hyphal mantle composed of interwoven hyphae branching from the spore wall; hyphae with or without terminal or intercalary vesiculate swellings. Subtending hypha with a wall concolorous or slightly lighter colored than the spore wall. Subtending hyphal wall layers continuous with spore wall layers, except for the innermost spore wall layer (Fig. 2B–D). Spore pore closed by a septum continuous with the penultimate spore wall layer and/or an invagination of the innermost spore wall layer (Fig. 2B–D), rarely open. Mantle and spores do not react in Melzer’s reagent. Germination by a germ tube penetrating through the outermost spore wall layer or the subtending hyphal lumen.

##### Ecology and distribution.

*Paracorymbiglomusglobiferum* was found in the field among roots of *Ammophilabreviligulata* Fern. and *Uniolapaniculata* L. that colonized coastal dunes at Cape May (New Jersey), Florida, near the city of Galinhos, and in the RDSE Ponta do Tubarão, Brazil (Koske and Walker 1986; Sylvia 1986; Sylvia and Will 1988; Wu and Sylvia 1993; Błaszkowski et al. 2019). Our analyses with environmental sequences showed that *P.globiferum* was also found as an uncultured *Diversispora* species with sequences AB670090–AB670092 in roots of *Ixerisrepens* (L.) A. Gray sampled in the Tottrii sand dunes in Japan. However, none of the five environmental sequences named *Corymbiglomusglobiferum*PQ669927, PQ669961, PQ669984, PQ669985, and PQ670011, which were derived from roots of *Heveabrasiliensis* Muli. Arg. growing in Kerala, India, clustered with the original *P.globiferum* sequences (data not shown).

*Paracorymbiglomuspacificum* spores were originally extracted from the root zone of *A.arenaria* at the mouth of Lake Budi, a saline ecosystem periodically connecting with the Pacific Ocean, located near the municipality of Puerto Saavedra in La Araucanía Region (southern Chile; Medina et al. 2014). In public databases, there is no sequence > 96% similar to the sequences that were used for the original characterization of *P.pacificum*.

In summary, *P.globiferum* appears to have a wide distribution in the world, where it occurs rather rarely. In contrast, *P.pacificum* may be endemic to Chile, or the occurrence of this species is very rare in the world.

In single-species cultures, *P.globiferum* formed mycorrhiza with *U.paniculata* (Sylvia and Burks 1988). The ability of *P.pacificum* to form a mycorrhizal association is unknown.

#### ﻿ Corymbiglomus

Taxon classificationFungiDiversisporalesDiversisporaceae

(Błaszk. & Chwat) emend. Błaszk., Niezgoda & B.T.Goto

11BA0180-FF2D-55A5-9E9E-2E4D115B4634

Fig. 1 , Suppl. material 1

##### Etymology.

Latin, *Corymbiglomus*, referring to the corymbiform organization of spores in clusters of the fungus.

##### Type genus.

*Corymbiglomuscorymbiforme* (Błaszk.) Błaszk. & Chwat emend. Błaszk. Niezgoda & B.T. Goto

##### Basionym.

*Glomuscorymbiforme* Błaszk.

##### Diagnosis.

Differs from *Paracorymbiglomus*, the sister two-species genus with *P.globiferum* and *P.pacificum* (Fig. 1, Suppl. material 1), in (i) the site of initiation of formation of the innermost spore wall layer (Fig. 2), (ii) the composition of the subtending hyphal wall (Fig. 2), (iii) the mode of formation and morphology of the hyphal mantle covering the spore surface, and (iv) the nucleotide composition of sequences of the 45S nuc rDNA region (see “Discussion” for details).

##### Genus description.

Spores hypogeous, occurring in corymbiform clusters when formed from branched sporophores, rarely produced singly. Spores light- to dark-colored, globose to subglobose, 50–220 µm diam, sometimes ovoid to pyriform, with one subtending hypha. Subcellular structure of spores composed of one spore wall containing three layers (layers 1–3). Outermost spore wall layer 1 covered or not covered by a hyphal mantle. Spore wall layer 3 laminate, semi-flexible, hyaline, 0.5–5.8 µm thick, sometimes detached from the inner surface of spore wall layer 2 in crushed spores, except at the spore base, always closely adherent to the inner surface of spore wall layer 2 at the spore base, continuous with the innermost subtending hyphal wall layer 3 at and well below the spore base (Fig. 2A). Hyphal mantle consisting of a network of hyphae branching dichotomously three to four times at more or less right angles; hyphae developing from spore wall layer 1; mantle frequently detached from mature spores. Subtending hypha with a wall concolorous or slightly lighter colored than the spore wall. Subtending hyphal wall layers continuous with spore wall layers 1–3 (Fig. 2A). The spore pore occluded by (i) ingrowth of spore wall layer 3, (ii) a septum continuous with the innermost laminae of spore wall layer 2, (iii) both the structures, and occasionally (iv) thickening of spore wall layer 2, rarely open. Mantle and spores do not react in Melzer’s reagent. Germination by a germ tube penetrating through the lumen of the subtending hypha.

##### Ecology and distribution.

In the field, *C.corymbiforme* was found in mixtures of rhizosphere soil and root fragments of numerous plant species inhabiting coastal and inland dunes of Spain, Poland, and Turkey (Błaszkowski et al. 2019). This fungus also occurred in semiarid open sandy grasslands of Hungary (Takacs and Bratek 2006; Błaszkowski, unpubl. data). Of the 43 45S environmental sequences (e.g., PQ669883, PQ669914, PQ669932, PQ670025) shown by BLASTn to represent *C.corymbiforme*, none clustered with our *C.corymbiforme* sequences (Fig. 1, Suppl. material 1) used in analyses aimed at a broader understanding of the global distribution of this species (data not shown). Thus, *C.corymbiforme* probably has a wide distribution in the world but occurs rather rarely. In single-species cultures, *C.corymbiforme* formed mycorrhiza with arbuscules and intra- and extraradical hyphae staining moderately to intensively in 0.1% Trypan blue (see fig. 4a, b in Błaszkowski et al. 2019).

#### ﻿ Diversispora conica

Taxon classificationFungiDiversisporalesDiversisporaceae

Błaszk., Niezgoda & B.T.Goto sp. nov.

0C3DD6CA-86B2-57E0-9D18-7698CE0867AE

858392

Fig. 3A–H

##### Specimens examined.

Greece. Spores from three single-species cultures established from spores extracted from trap cultures inoculated with rhizosphere soil and root fragments of *Ammophilaarenaria* from a maritime sand dune site of the beach Voidokoilia (36°57'N, 21°39'E), the Peloponnese Peninsula, Greece, September 8, 2015, J. Błaszkowski (***holotype*** slide with spores no. ZTMyc 0067489, ***isotypes*** slides with spores no. 3997–4004, LPPDSE).

##### Etymology.

Latin, *conica*, referring to the conical fragments of spore wall layer 4 associated with spore wall layer 3 in crushed spores.

##### Diagnosis.

Differs from (a) *D.aestuarii*, the close phylogenetic relative (Fig. 1, Suppl. material 1) in (i) phenotypic properties of spore wall layer 4, (ii) morphometric characters of spores and spore wall layer 1, and (iii) nucleotide composition of sequences of the 45S nuc rDNA region, (b) *D.nevadensis*, another close phylogenetic relative (Fig. 1, Suppl. material 1), in (i) the mode of spore formation, (ii) the spore subcellular structure, and (iii) nucleotide composition of sequences of the 45S nuc rDNA region (see “Discussion” for details).

##### Description.

**Figure 3.**
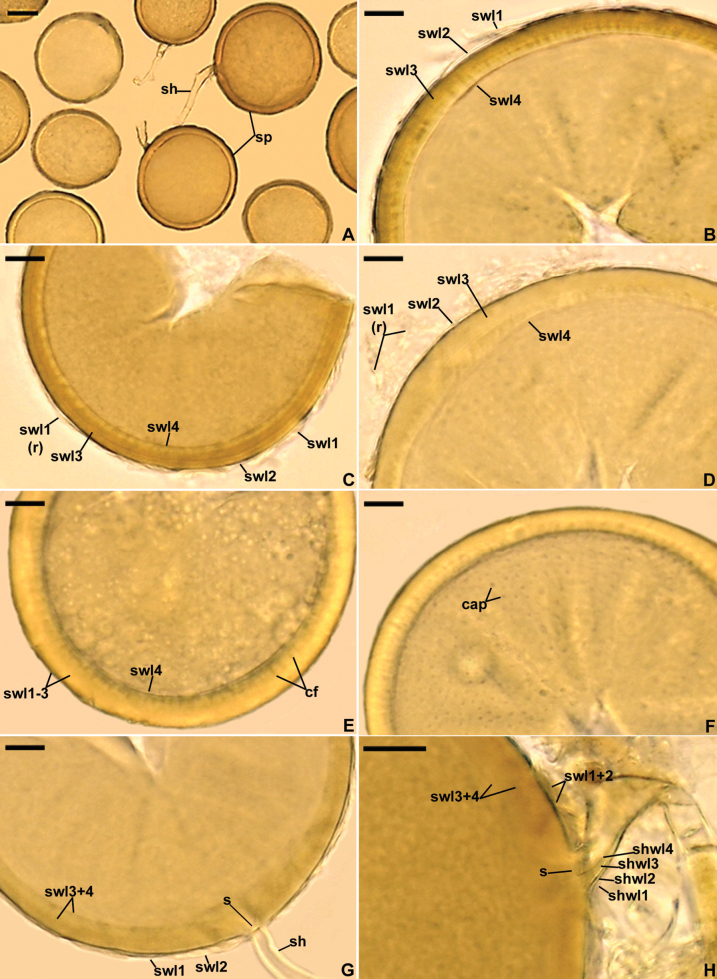
*Diversisporaconica***A** intact spores (sp) with subtending hyphae (sh) **B–D** spore wall layers (swl) 1–4 (r = remnants) **E** spore wall layers (swl) 1–4; conical fragments (cf) of swl4 associated with the lower surface of the laminate layer 3 viewed in cross-section **F** circular attachment points (cap) of spore wall layer 4 to the laminate layer 3 seen in plan view **G** spore wall layers (swl) 1–4, septum (s) occluding the space between the subtending hyphal lumen and the spore interior, and subtending hypha (sh) **H** subtending hyphal wall layers (shwl) 1–4 continuous with spore wall layers (swl) 1–4 and septum (s) occluding the spore pore at the spore base **A** spores in lactic acid **B, C, D, F** spores in PVLG**E, G, H** spores in PVLG+Melzer’s reagent **A** light microscopy **B–H** differential interference microscopy. Scale bars: 20 μm (**A**); 10 μm (**B–H**).

Glomerospores formed singly in soil, arise blastically at tips of subtending hyphae (Fig. 3A, G, H). Spores pale yellow (4A3) to brownish orange (5C5); globose to subglobose; (55–)81(–114) µm diam; rarely ovoid; 111–115 × 118–133 µm; with one subtending hypha (Fig. 3A, G, H). Spore wall composed of four layers (Fig. 3B–E, G, H). Layer 1, forming the spore surface, evanescent, flexible, hyaline, (0.6–)0.9(–1.3) µm thick, when thin difficult to see, usually short-lived, and completely sloughed off in most mature spores (Fig. 3B–E, G, H). Layer 2 permanent, uniform (without visible sublayers), smooth, semi-rigid, hyaline, (0.8–)1.2(–1.8) µm thick, tightly adherent to the upper surface of layer 3 (Fig. 3B–E, G, H). Layer 3 laminate, smooth, semi-rigid, pale yellow (4A3) to brownish orange (5C5), (4.6–)6.7(–11.2) µm thick, consisting of very thin, < 0.5 µm thick, laminae, tightly adherent to and not separating from each other in even vigorously crushed spores (Fig. 3B–E, G, H). Layer 4 uniform, flexible to semi-flexible, hyaline, (0.8–)1.0(–1.6) µm thick, usually easily separating from the lower surface of layer 3 in young and freshly mature spores, except for small fragments evenly distributed on its upper surface, which remain associated with layer 2; then these fragments resemble cones and circles when viewed in cross-section and plan view, respectively (Fig. 3B–H); in older spores, layer 4 usually tightly adheres to layer 3 (Fig. 3G, H). None of spore wall layers 1–4 stains in Melzer’s reagent (Fig. 3G, H). Subtending hypha hyaline to greyish yellow (4B3); straight or recurved, cylindrical or slightly constricted at the spore base; (3.4–)7.5(–11.4) µm wide at the spore base (Fig. 3A, G, H); not breaking in crushed spores. Wall of subtending hypha hyaline; (1.0–)2.6(–3.5) µm thick at the spore base; consisting of four layers continuous with spore wall layers 1–4 (Fig. 3G, H). Pore (1.0–)2.5(–7.6) µm wide at the spore base, open or occluded by a straight or slightly curved septum, 1.0–1.2 µm thick, continuous with some outermost laminae of spore wall layer 3 (Fig. 3G, H). Spore content of hyaline oily substance. Germination unknown.

##### Ecology and distribution.

In the field, *D.conica* probably formed a mycorrhizal association with *A.arenaria*, which inhabited the Mediterranean dunes of the beach Voidokoilia (36°57'N, 21°39'E) on the Peloponnese Peninsula, Greece. However, no molecular analyses were performed to confirm this assumption. Our phylogenetic analyses with environmental sequences shown by BLASTn to be > 96% similar to the *D.conica* sequences suggested that this species was also found in roots of *Acerplatanoides* L., *Populustremula* L., and *Zeamays* L. sampled in Melnik, Central Bohemia, Czech Republic (sequences HG425548, HG425551, HG425553). In single-species cultures, *D.conica* sporulated abundantly and formed typical mycorrhiza with arbuscules and intra- and extraradical hyphae. No vesicles were observed.

## ﻿Discussion

The analyses and comparisons conducted in this study resolved uncertainties regarding the morphological similarities and phylogenetic relationships of three *Corymbiglomus* species and their relationships to *Redeckera* species (see “Introduction”). Furthermore, these studies confirmed our initial recognition that Isolate 449 belongs to the genus *Diversispora* and proved that it is a new species, here described as *D.conica*.

The main morphological character that convincingly supported the decision to transfer *C.globiferum* and *C.pacificum* to a new genus, here described as *Paracorymbiglomus* with *P.globiferum* comb. nov. and *P.pacificum* comb. nov., was the location of the innermost spore wall layer 3 relative to the spore subtending hyphal wall at and below the spore base in these two species as compared to *C.corymbiforme* (Fig. 2A–D). This location was clearly shown in the INVAM characterization of *P.globiferum* (considered as *D.globifera*; https://invam.ku.edu/), which was omitted in earlier studies of *Corymbiglomus* species (Błaszkowski 2012; Błaszkowski et al. 2019). According to this characterization, *P.globiferum* spore wall layer 3 starts to form at most half the thickness of the laminate spore wall layer 2, where it maintains physical contact with this layer even in crushed spores, and does not occur as a component in the spore subtending hyphal wall (Fig. 2B, C). This mode of formation and location was also given in the description of *P.pacificum* (Medina et al. 2014) and is depicted in our Fig. 2D. However, Medina et al. (2014) called the innermost component of the spore subcellular structure an inner wall arising de novo.

One of the Diversisporales species producing glomoid spores sensu Morton and Redecker (2001) with two spore walls, of which spore wall 2 arises de novo, is *Pacisporascintillans*. However, spore wall 2 of *P.scintillans* arises after the full differentiation of spore wall 1, which forms the spore surface, and has no physical contact with either spore wall 1 (vs. it has in *C.corymbiforme*, *P.globiferum*, and *P.pacificum*) or the spore subtending hyphal wall (vs. it has in *C.corymbiforme*; see figs 3d, f, g, h, 4f, 5c, d, f in Błaszkowski et al. 2019 and Fig. 2A–D). We, therefore, accepted the conclusion of Stürmer and Morton (1997) on the innermost spore wall layer of *E.claroidea*, which resulted from ontogenetic studies of this species, and also treat this innermost component of the subcellular structure of *C.corymbiforme*, *P.globiferum*, and *P.pacificum* spores as a component of one structure, the spore wall. Furthermore, we concluded that this component of the spore wall in *P.globiferum* and *P.pacificum* is formed in the last stage of the spore wall differentiation. In *C.corymbiforme*, on the other hand, all three layers of the spore subtending hyphal wall are continuous with spore wall layers 1–3 (see figs 3d, f, g, h in Błaszkowski et al. 2019 and Fig. 2A), develop simultaneously, and this development is usually initiated well below the spore base (Błaszkowski 2012; Błaszkowski et al. 2019).

The creation of *Paracorymbiglomus* with *P.globiferum* and *P.pacificum* was equally convincingly supported by our phylogenetic analyses and sequence comparisons of these two species with each other and relative to *C.corymbiforme*. *Corymbiglomuscorymbiforme* was placed in an autonomous clade sister to the clade with *P.globiferum* and *P.pacificum*, and each of these clades received full BI and ML supports (Fig. 1, Suppl. material 1). Importantly, no analysis supported the node connecting the *C.corymbiforme* clade with the *C.globiferum* plus *C.pacificum* clade. The divergences of the 45S sequences of *C.corymbiforme* from those of *P.globiferum* and *P.pacificum* were 11.6–15.1%, which was similar to the range of sequence divergences between *C.corymbiforme* vs. *R.megalocarpum* (10.4–12.8%) and other Glomeromycota genera (da Silva et al. 2024, 2025). In contrast, the 4.4–5.5% divergences between *P.globiferum* and *P.pacificum* were within the interspecific sequence differences of members of this phylum (Błaszkowski et al. 2022a, 2023; Crossay et al. 2024).

Unlike in Tedersoo et al. (2024), all our phylogenetic analyses and sequence comparisons clearly separated *Corymbiglomus* and *Paracorymbiglomus* from *Redeckera* (Fig. 1, Suppl. materials 1, 2) and suggested that the species composition of *Redeckera* is unclear. This assumption was based on the inconsistent placement of *R.fulvum* and *R.pulvinatum* relative to the clade with *R.megalocarpum* in both the 45S+*rpb1* and 45S trees, which would indicate that these species are not related within the genus *Redeckera*. Most importantly, this thesis was strongly supported by the enormous divergences of the 18S-ITS sequences of *R.fulvum* and *R.pulvinatum* from the 18S-ITS sequences of *R.megalocarpum*, amounting to about 40%. This suggested that *R.fulvum* and *R.pulvinatum* either do not belong to *Redeckera*, despite their morphological and ecological similarities, or the phylogenetic membership of these species in *Redeckera* is encoded in other genes, e.g., 28S.

*Corymbiglomus* and *Paracorymbiglomus* species also differ morphologically and ecologically substantially from *R.megalocarpum*, the type species of *Redeckera* (Schüßler and Walker 2010). The morphological differences mainly lie in the composition of the spore wall and subtending hyphal wall, and the features of the septum closing the spore pore (Redecker et al. 2007; Oehl et al. 2011a). In *R.megalocarpum*, the spore wall and subtending hyphal wall consist of two layers (vs. three layers), and the septum resembles a straight and wide bridge connecting the main structural laminate spore wall layer 2 when viewed in cross-section (vs. it is curved, recessed into the spore subtending hyphal lumen, and continuous with spore wall layer 3). Moreover, in *R.megalocarpum*, the thickness of this septum is similar to the thickness of the laminate spore wall layer 2, which is much thinner than the main structural laminate spore wall layer 2 of *Corymbiglomus* and *Paracorymbiglomus* species and other Diversisporaceae species producing diversisporoid spores (Schüßler et al. 2011; Błaszkowski et al. 2022a).

All species assigned to *Redeckera* were described to produce spores in compact epigeous glomerocarps, and none of these species has been known to form mycorrhizae (Redecker et al. 2007). *Corymbiglomus* and *Paracorymbiglomus* species produced hypogeous spores in loose clusters and singly, and *C.corymbiforme* and *C.globiferum* formed mycorrhiza in single-species cultures; the latter property is unknown for *C.pacificum* (Sylvia and Burks 1988; Błaszkowski 2012; Błaszkowski et al. 2019).

Regarding the terminology of spore names, the composition of the spore subtending hyphal wall and the spore wall of *C.corymbiforme* indicates that these spores should be called glomoid sensu Morton and Redecker (2001). In contrast, the spore characters of *Paracorymbiglomus* correspond to those of diversisporoid sensu Oehl et al. (2011a, b).

Our BI and ML phylogenetic analyses fully supported the novelty of *D.conica* (Fig. 1, Suppl. material 1). However, they left ambiguity regarding the closest relative of this new species. The analyses based on 45S+*rpb1* and 45S sequences showed that the sisters of *D.conica* were *D.aestuarii* and *D.bareae*, respectively (Fig. 1, Suppl. material 1). However, neither analysis supported the node connecting *D.conica* with these indicated relatives.

Morphologically, *D.conica* strongly resembles *D.aestuarii*. Both species produce diversisporoid spores; the color, composition of the spore wall, and properties of the spore subtending hypha are similar (Błaszkowski et al. 2022a). The major difference readily separating these two species is the phenotypic property of the innermost spore wall layer 4. The localized/punctate detachment of this smooth, unsculptured layer from the lower surface of the laminate spore wall layer 3 in crushed spores gives the erroneous impression that the upper surface of layer 4 is covered with densely and evenly distributed conical outgrowths when viewed in cross-section (Fig. 3B–E). This property has not been observed so far in spores of any member of Glomeromycota. Moreover, compared to *D.aestuarii*, globose spores of *D.conica* are 1.2–1.4-fold smaller, and their spore wall layer 1 is 2.0–5.4-fold thinner. Finally, the distinctiveness of *D.conica* from *D.aestuarii* was proven by the strong divergences of their 45S sequences, which were 5.6–6.2%.

There is no morphological similarity between *D.conica* and *D.bareae. Diversisporabareae*, originally described as *Otosporabareae* (Palenzuela et al. 2008), produces spores laterally on the neck of a sporiferous saccule, and the spores have two walls. Oehl et al. (2011b) named such spores otosporoid. The 3.8–4.6% differences between the 45S sequences of *D.aestuarii* and *D.bareae* and the presence of *D.nevadensis*, originally described as *Entrophosporanevadensis* (Palenzuela et al. 2010) due to its similar mode of spore formation to that of *E.infrequens*, the type species of the genus *Entrophospora* (Schüßler and Walker 2010; Błaszkowski et al. 2022b), among *Diversispora* species suggest that members of *Diversispora* are polymorphic fungi, with the potential ability to produce diversisporoid, tricisporoid, and otosporoid spores sensu Oehl et al. (2011a, b).

## Supplementary Material

XML Treatment for Paracorymbiglomus

XML Treatment for Corymbiglomus

XML Treatment for Diversispora conica

## ﻿Additional information

### Author ORCIDs

Janusz Błaszkowski https://orcid.org/0000-0003-3688-164X

Bruno Tomio Goto https://orcid.org/0000-0001-6157-4954

Szymon Zubek https://orcid.org/0000-0003-1338-9572

Paweł Milczarski https://orcid.org/0000-0003-2622-8938

Ryszard Malinowski https://orcid.org/0000-0003-3413-2653

Piotr Niezgoda https://orcid.org/0000-0002-0560-6154

Tomasz Błaszkowski https://orcid.org/0000-0003-4987-2750

### Data availability

All of the data that support the findings of this study are available in the main text or Supplementary Information.

## Supplementary materials

Supplementary material 1 Supplementary information 1 Data type zip Explanation note 50% majority-rule consensus tree from the Bayesian analysis of the 45S alignment with sequences of *Diversisporaconica* (= Isolate 449), *Corymbiglomuscorymbiforme*, *Paracorymbiglomusglobiferum*, *P.pacificum*, 32 other species of Diversisporaceae, as well as *Acaulosporalaevis*, *Pacisporascintillans*, and *Sacculosporabaltica* serving as outgroup. The new species and genus are in bold font. The Bayesian posterior probabilities ≥ 0.90 and ML bootstrap values ≥ 50% are shown near the branches, respectively. Bar indicates a 0.05 expected change per site per branch. https://binary.pensoft.net/file/1322763This dataset is made available under the Open Database License (http://opendatacommons.org/licenses/odbl/1.0/). The Open Database License (ODbL) is a license agreement intended to allow users to freely share, modify, and use this Dataset while maintaining this same freedom for others, provided that the original source and author(s) are credited.Author: Janusz Błaszkowski

Supplementary material 2 Supplementary information 2 Data type zip Explanation note 50% majority-rule consensus tree from the Bayesian analysis of the *rpb1* alignment with sequences of *Corymbiglomuscorymbiforme*, 21 other species of Diversisporaceae, as well as *Acaulosporalaevis* and *Sacculosporabaltica* serving as outgroup. The Bayesian posterior probabilities ≥ 0.90 and ML bootstrap values ≥ 50% are shown near the branches, respectively. Bar indicates a 0.05 expected change per site per branch. https://binary.pensoft.net/file/1322764This dataset is made available under the Open Database License (http://opendatacommons.org/licenses/odbl/1.0/). The Open Database License (ODbL) is a license agreement intended to allow users to freely share, modify, and use this Dataset while maintaining this same freedom for others, provided that the original source and author(s) are credited.Author: Janusz Błaszkowski
