# ﻿Corrigendum: Błaszkowski J, Goto BT, Zubek Sz, Milczarski P, Malinowski R, Niezgoda P, Błaszkowski T (2025) *Paracorymbiglomus* gen. nov., *Diversispora conica* sp. nov., and new combinations in Diversisporaceae (Glomeromycota). MycoKeys 117: 171–190. https://doi.org/10.3897/mycokeys.117.148052

**DOI:** 10.3897/mycokeys.124.171105

**Published:** 2025-11-13

**Authors:** Janusz Błaszkowski, Bruno Tomio Goto, Szymon Zubek, Paweł Milczarski, Ryszard Malinowski, Piotr Niezgoda, Tomasz Błaszkowski

**Affiliations:** 1 Department of Environmental Management, West Pomeranian University of Technology in Szczecin, Słowackiego 17, PL–71434 Szczecin, Poland; 2 Departamento de Botânica e Zoologia, Universidade Federal do Rio Grande do Norte, Campus Universitário, 59078–900, Natal, RN, Brazil; 3 Institute of Botany, Faculty of Biology, Jagiellonian University, 30–387, Kraków, Poland; 4 Department of Genetic, Plant Breeding & Biotechnology, West Pomeranian University of Technology in Szczecin, Słowackiego 17, PL–71434 Szczecin, Poland; 5 Department of General and Oncological Surgery, Pomeranian Medical University in Szczecin, Szczecin, Poland

## ﻿

We have been informed that the names *Paracorymbiglomus* and *Paracorymbiglomus pacificum* are not valid due to the erroneous use of the “Type genus” instead of the “Type species” of *Paracorymbiglomus* and incomplete descriptions of the basionyms *Glomus globiferum* and *Corymbiglomus pacificum* (Shenzhen code: Art. 40.1 and 41.5, see Arts 40.3 and Arts 6.3, 12.1; Turland et al. 2018). The correct data for these taxa are as follows.

### ﻿ Paracorymbiglomus

Taxon classificationFungi

Błaszk., Niezgoda & B.T. Goto. gen. nov.

9DD1879F-8CDF-5073-97AD-05DBCD90EE2C

858391

#### Type species.

*Paracorymbiglomus globiferum* (Koske & C. Walker) Błaszk., Niezgoda & B.T.Goto comb. nov. MycoBank No: 858393.

#### Basionym.

*Glomus globiferum* Koske & C. Walker, Mycotaxon 26. 133. 1986.

#### Other species.

*Paracorymbiglomus pacificum* (Oehl, J. Medina, P. Cornejo, Sánchez-Castro, G.A. Silva & Palenz.) Błaszk., Niezgoda & B.T.Goto comb. nov. MycoBank No: 858394.

***Basionym***: *Corymbiglomus pacificum* Oehl, J. Medina, P. Cornejo, Sánchez-Castro, G.A. Silva & Palenz., Mycotaxon 127. 176. 2014. MycoBank No: 805601.

## Supplementary Material

XML Treatment for Paracorymbiglomus

## ﻿Additional information

### Use of AI

No use of AI was reported.

### Author ORCIDs

Janusz Błaszkowski https://orcid.org/0000-0003-3688-164X

Bruno Tomio Goto https://orcid.org/0000-0001-6157-4954

Szymon Zubek https://orcid.org/0000-0003-1338-9572

Paweł Milczarski https://orcid.org/0000-0003-2622-8938

Ryszard Malinowski https://orcid.org/0000-0003-3413-2653

Piotr Niezgoda https://orcid.org/0000-0002-0560-6154

Tomasz Błaszkowski https://orcid.org/0000-0003-4987-2750

### Data availability

All of the data that support the findings of this study are available in the main text.
