# Corrigendum: Błaszkowski J, Goto BT, Zubek S, Milczarski P, Malinowski R, Niezgoda P, Błaszkowski T (2025) *Paracorymbiglomus* gen. nov., *Diversispora conica* sp. nov., and new combinations in Diversisporaceae (Glomeromycota). MycoKeys 117: 171–190. https://doi.org/10.3897/mycokeys.117.148052

**DOI:** 10.3897/mycokeys.134.198487

**Published:** 2026-06-18

**Authors:** Janusz Błaszkowski, Bruno Tomio Goto, Szymon Zubek, Paweł Milczarski, Ryszard Malinowski, Piotr Niezgoda, Tomasz Błaszkowski

**Affiliations:** 1 Department of Environmental Management, West Pomeranian University of Technology in Szczecin, Słowackiego 17, PL–71434 Szczecin, Poland Department of General and Oncological Surgery, Pomeranian Medical University in Szczecin Szczecin Poland; 2 Departamento de Botânica e Zoologia, Universidade Federal do Rio Grande do Norte, Campus Universitário, 59078–900, Natal, RN, Brazil Institute of Botany, Faculty of Biology, Jagiellonian University Kraków Poland; 3 Institute of Botany, Faculty of Biology, Jagiellonian University, 30–387, Kraków, Poland Departamento de Botânica e Zoologia, Universidade Federal do Rio Grande do Norte, Campus Universitário Natal Brazil; 4 Department of Genetic, Plant Breeding & Biotechnology, West Pomeranian University of Technology in Szczecin, Słowackiego 17, PL–71434 Szczecin, Poland Department of Environmental Management, West Pomeranian University of Technology in Szczecin Szczecin Poland; 5 Department of General and Oncological Surgery, Pomeranian Medical University in Szczecin, Szczecin, Poland Department of Genetic, Plant Breeding & Biotechnology, West Pomeranian University of Technology in Szczecin Szczecin Poland

We have been informed that the names *Paracorymbiglomus* and *Paracorymbiglomus pacificum* are not validly published. Full and direct references to the basionym names *Glomus globiferum* and *Corymbiglomus pacificum* were omitted which rendered the names of the new combinations invalid and also that of the generic name (Madrid code: Art. 40.1, see Arts 40.3 and Arts 6.3, 12.1 and Art. 41.5; Turland et al. 2025). The names are validated herein.

## Paracorymbiglomus

Taxon classificationFungiDiversisporalesDiversisporaceae

Błaszk., P. Niezgoda & B.T. Goto gen. nov.

D1A9CFA3-BCC3-5BE8-8E30-B607F96F0638

863198

### Note.

For a detailed description see Błaszkowski et al., MycoKeys 117: 178 (2015).

### Type species.

*Paracorymbiglomus globiferum* (Koske & C. Walker) Błaszk., P. Niezgoda & B.T. Goto, comb. nov. MB 863199.

### Basionym.

*Glomus globiferum* Koske & C. Walker, Mycotaxon 26: 133 (1986).

*Paracorymbiglomus pacificum* (Oehl, J. Medina, P. Cornejo, Sánchez-Castro, G.A. Silva & Palenz.) Błaszk., P. Niezgoda & B.T. Goto, comb. nov. MB 863201.

**Basionym**. *Corymbiglomus pacificum* Oehl, J. Medina, P. Cornejo, Sánchez-Castro, G.A. Silva & Palenz., Mycotaxon 127: 176 (2014).

## Supplementary Material

XML Treatment for Paracorymbiglomus

## Additional information

### Artificial Intelligence (AI) use

The authors accept full responsibility for the content of the manuscript, including the disclosure of any use of AI.

No AI tools were used in the preparation of this manuscript.

### Author ORCIDs

J. Błaszkowski https://orcid.org/0000-0003-3688-164X

B.T. Goto https://orcid.org/0000-0001-6157-4954

S. Zubek https://orcid.org/0000-0003-1338-9572

P. Milczarski https://orcid.org/0000-0003-2622-8938

R. Malinowski https://orcid.org/0000-0003-3413-2653

P. Niezgoda https://orcid.org/0000-0002-0560-6154

T. Błaszkowski https://orcid.org/0000-0003-4987-2750

### Data availability

All of the data that support the findings of this study are available in the main text.
