# Supplementary figures and images for: ﻿Paracorymbiglomus gen. nov., Diversisporaconica sp. nov., and new combinations in Diversisporaceae (Glomeromycota)
Source: MycoKeys. 2025 May 5;117:171–90. doi: 10.3897/mycokeys.117.148052 (PMC12070055; doi:10.3897/mycokeys.117.148052)

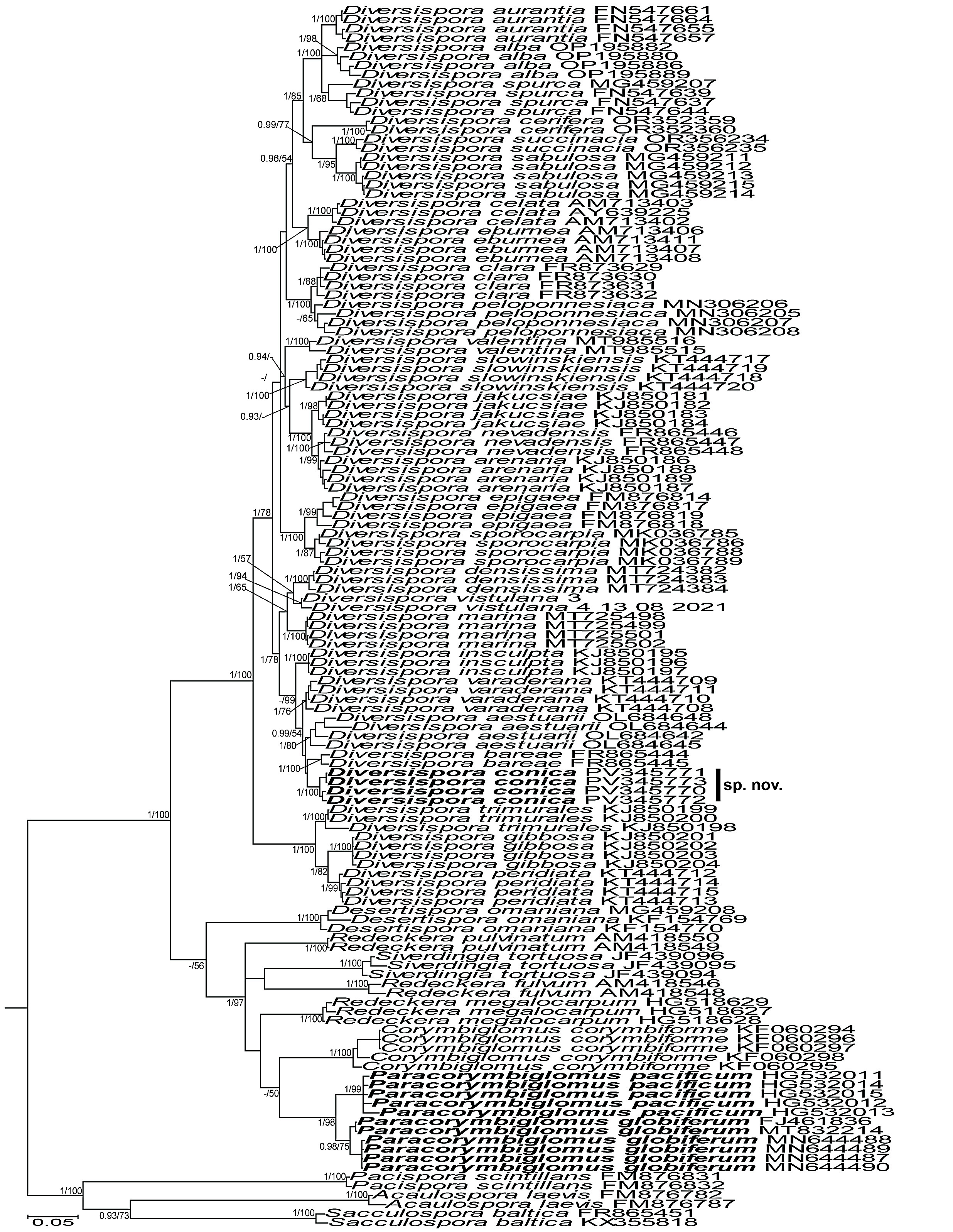

Supplement: Supplementary material 1 — Supplementary information 1 [file mycokeys-117-171-s001.zip › 148052_1C-1-A_Revised_Supplementary_material_1.jpg]

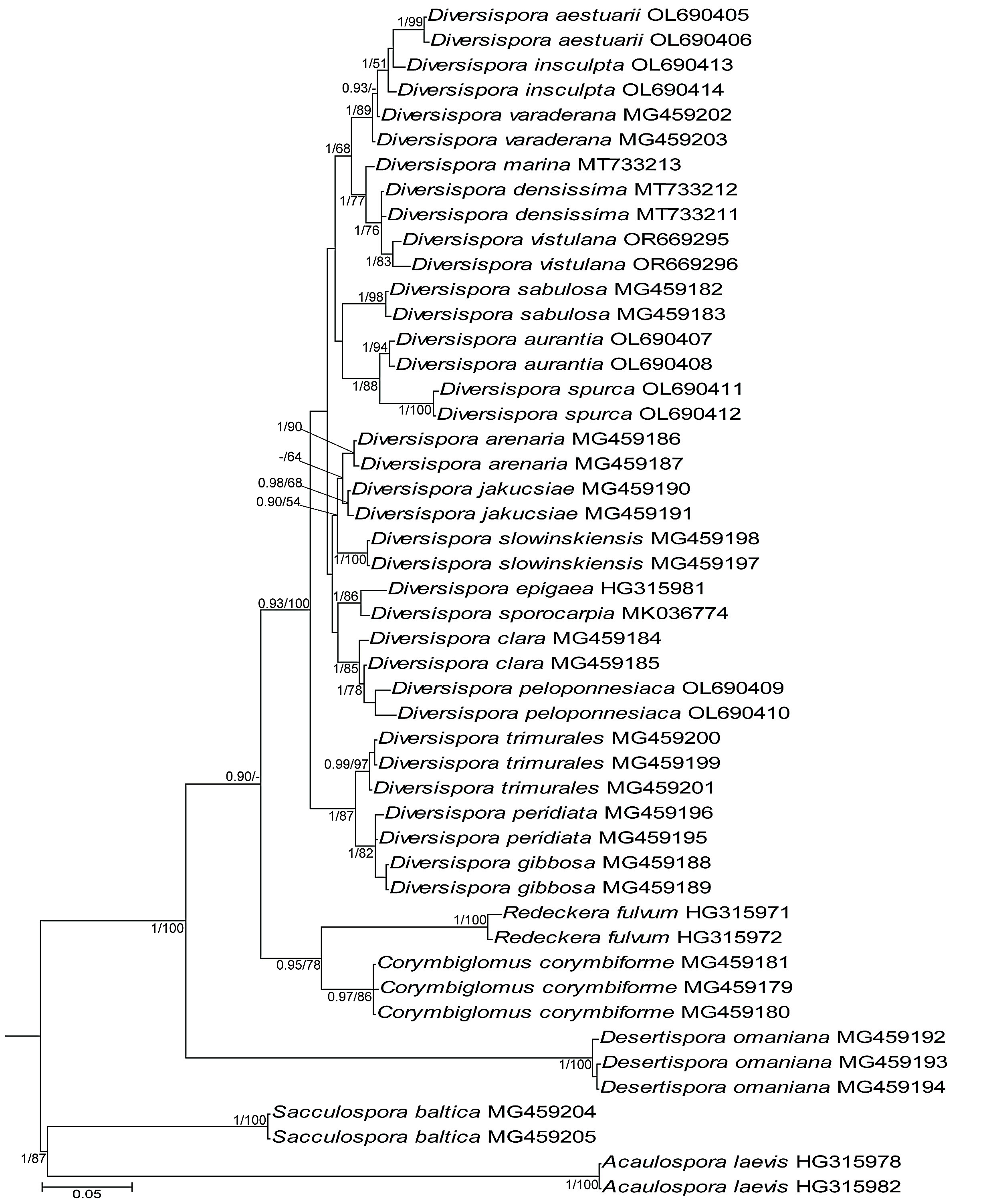

Supplement: Supplementary material 2 — Supplementary information 2 [file mycokeys-117-171-s002.zip › 148052_1C-1-A_Revised_Supplementary_material_2.jpg]
